# P-348. Real-world efficacy, safety and persistence of dolutegravir/lamivudine vs. bictegravir/emtricitabine/tenofovir alafenamide among virologically suppressed adults with HIV-results from the 96-week observational extension phase of the DYAD study

**DOI:** 10.1093/ofid/ofaf695.566

**Published:** 2026-01-11

**Authors:** Charlotte-Paige M Rolle, Jamie Castano, Vu Nguyen, Federico Hinestrosa, Edwin DeJesus

**Affiliations:** Orlando Immunology Center ; Emory Rollins School of Public Health, Orlando, FL; Orlando Immunology Center, Orlando, Florida; Orlando Immunology Center, Orlando, Florida; Orlando Immunology Center, University of Central Florida College of Medicine, Orlando, FL; Orlando Immunology Center, University of Central Florida College of Medicine, Orlando, FL

## Abstract

**Background:**

DYAD demonstrated noninferior efficacy of switching to dolutegravir/lamivudine (DTG/3TC) vs. continuing bictegravir/emtricitabine/tenofovir alafenamide (B/F/TAF) among virologically suppressed adults through Week (W) 48. Here, we present real-world efficacy, safety and persistence from the 96-week observational extension phase.

**Figure:**
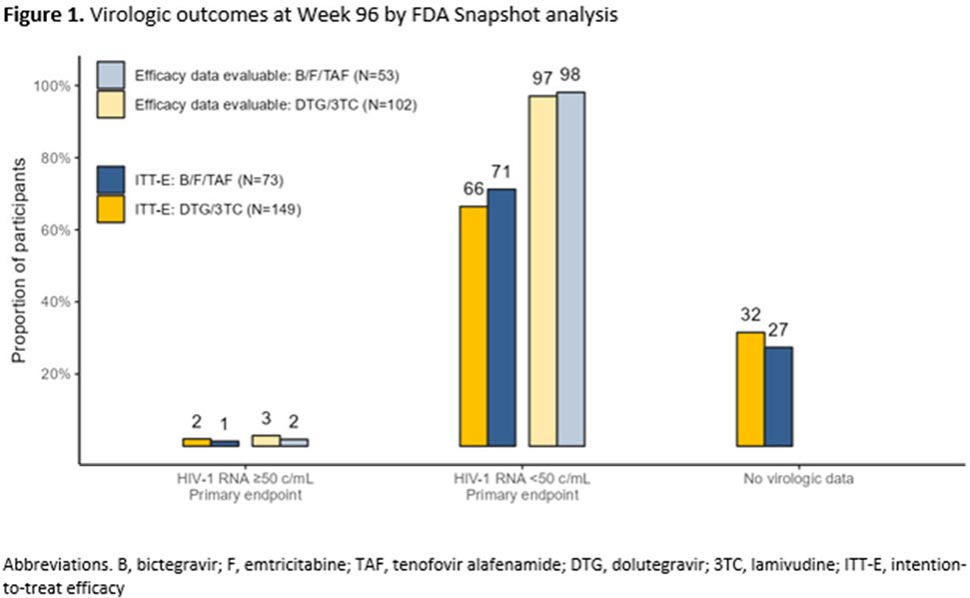

**Figure:**
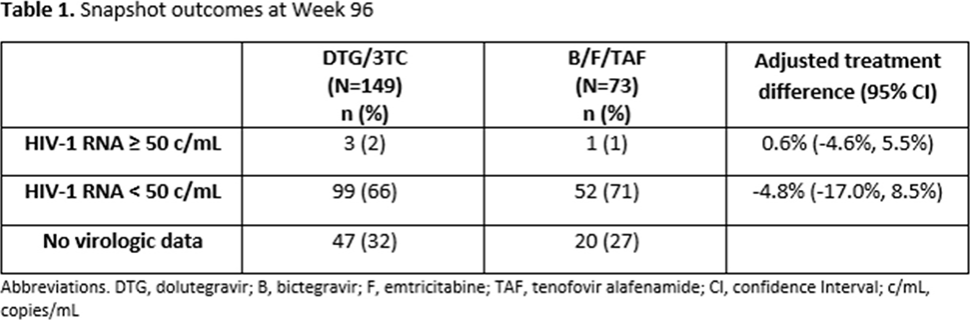

**Methods:**

DYAD (NCT04585737) was an open-label clinical trial that randomized adults with HIV-1 RNA<50 copies/mL and no prior virologic failure (2:1) to switch to DTG/3TC vs. continue B/F/TAF. Primary endpoint was the proportion with HIV-1 RNA≥50 copies/mL at W48. At study exit, consent was obtained from participants to collect and report virologic efficacy, safety and persistence on therapy from medical records through W96 and W144.

**Results:**

Among 222 enrolled, DYAD randomized 149 to switch to DTG/3TC and 73 to continue B/F/TAF, 134 on DTG/3TC and 65 on B/F/TAF completed W48. At study exit, 124 on DTG/3TC and 63 on B/F/TAF consented to participate in the observational extension phase. At W96, 3 (2%) on DTG/3TC and 1 (1%) on B/F/TAF had HIV-1 RNA≥50 c/mL (adjusted treatment difference 0.6%, 95% confidence interval [-4.6%, 5.5%]). HIV-1 RNA< 50 copies/mL was observed in 99 (66%) on DTG/3TC and 52 (71%) on B/F/TAF (adjusted treatment difference -4.8%, 95% confidence interval [-17.0%, 8.5%]). Through W96, 13 on DTG/3TC and 6 on B/F/TAF met confirmed virologic failure criteria, resistance was observed in 2 on DTG/3TC and 1 on B/F/TAF. At W96, 112 (75%) persisted on DTG/3TC and 59 (80%) persisted on B/F/TAF. The most common reason for DTG/3TC discontinuation was adverse events (AEs) in 11/37, whereas B/F/TAF discontinuation was most commonly due to consent withdrawal in 5/14. Drug-related AEs occurred in 42 (28%) on DTG/3TC and 4 (5%) on B/F/TAF through W96. No significant differences in mean change from baseline in creatinine, lipid parameters, weight, and BMI were observed between treatment groups at W96.

**Conclusion:**

In the 96-week DYAD observational extension phase, there was no significant difference in virologic efficacy among those switching to DTG/3TC vs. continuing B/F/TAF and no new cases of resistance between W48-96. Persistence was similar between treatment arms, however drug-related AEs and discontinuations due to AEs were higher with DTG/3TC.

**Disclosures:**

Charlotte-Paige M. Rolle, MD, MPH, Gilead Sciences: Grant/Research Support|Gilead Sciences: Honoraria|MSD: Grant/Research Support|ViiV Healthcare: Advisor/Consultant|ViiV Healthcare: Grant/Research Support|ViiV Healthcare: Honoraria Federico Hinestrosa, MD, Gilead Sciences: Honoraria|MSD: Honoraria

